# Friends-Enemies: Endogenous Retroviruses Are Major Transcriptional Regulators of Human DNA

**DOI:** 10.3389/fchem.2017.00035

**Published:** 2017-06-08

**Authors:** Anton A. Buzdin, Vladimir Prassolov, Andrew V. Garazha

**Affiliations:** ^1^Department of Cell Biology, Engelhardt Institute of Molecular Biology, Russian Academy of SciencesMoscow, Russia; ^2^Centre for Convergence of Nano-, Bio-, Information and Cognitive Sciences and Technologies, National Research Centre “Kurchatov Institute,”Moscow, Russia; ^3^Group for Genomic Regulation of Cell Signaling Systems, Shemyakin-Ovchinnikov Institute of Bioorganic ChemistryMoscow, Russia; ^4^Department of Biomedicine, Moscow Institute of Physics and TechnologyMoscow, Russia

**Keywords:** retrovirus, gene expression regulation, pathology, cancer, inflammation, stress, stability, infection

## Abstract

Endogenous retroviruses are mobile genetic elements hardly distinguishable from infectious, or “exogenous,” retroviruses at the time of insertion in the host DNA. Human endogenous retroviruses (HERVs) are not rare. They gave rise to multiple families of closely related mobile elements that occupy ~8% of the human genome. Together, they shape genomic regulatory landscape by providing at least ~320,000 human transcription factor binding sites (TFBS) located on ~110,000 individual HERV elements. The HERVs host as many as 155,000 mapped DNaseI hypersensitivity sites, which denote loci active in the regulation of gene expression or chromatin structure. The contemporary view of the HERVs evolutionary dynamics suggests that at the early stages after insertion, the HERV is treated by the host cells as a foreign genetic element, and is likely to be suppressed by the targeted methylation and mutations. However, at the later stages, when significant number of mutations has been already accumulated and when the retroviral genes are broken, the regulatory potential of a HERV may be released and recruited to modify the genomic balance of transcription factor binding sites. This process goes together with further accumulation and selection of mutations, which reshape the regulatory landscape of the human DNA. However, developmental reprogramming, stress or pathological conditions like cancer, inflammation and infectious diseases, can remove the blocks limiting expression and HERV-mediated host gene regulation. This, in turn, can dramatically alter the gene expression equilibrium and shift it to a newer state, thus further amplifying instability and exacerbating the stressful situation.

Human endogenous retroviruses (HERVs) and related genetic elements occupy ~8% of human genome. They are thought to be remnants of multiple ancient retroviral infections (Sverdlov, 2000; Belshaw et al., 2004; Buzdin, 2007). HERV insertions occurred in the ancestral germ cell lineage, fixed in the genome and became inheritable (Buzdin et al., 2003; Dewannieux and Heidmann, 2013). In the human DNA, HERVs are represented by 504 groups including 717.778 individual fragments (RepeatMasker, hg19). The individual HERV copies are frequently interrupted by other sequences, such as transposable elements, and may each represent two or more genomic fragments. Older HERVs have accumulated more mutations, including indels, and thus are more fragmented then the evolutionary young elements. For example, the MER-41-int element located at the position chr1:26,952,949-26,962,938 (hg19 assembly) is broken into four fragments in the genome, but biologically this was a single HERV.

Many families of HERVs are highly transcriptionally active in human tissues (Buzdin et al., 2006a; Maliniemi et al., 2013). Genomic copies of HERVs are of particular interest because in addition to viral genes they also have various regulatory sequences concentrated in their *long terminal repeats* (LTRs)—about 1 kb long fragments of DNA flanking the “body” of an element (Figure 1). The LTRs serve as promoters (Buzdin et al., 2006a), enhancers (Chuong et al., 2013; Suntsova et al., 2013), polyadenylation signals (Suntsova et al., 2015), chromatin folding reshapers (Schumann et al., 2010), and binding sites for various nuclear proteins (Young et al., 2013). Importantly, most of HERVs reside in the human genome as solitary LTRs arisen due to homologous recombinations between the two 5′- and 3′-flanking LTRs of the same full-length element (Hughes and Coffin, 2004). In turn, further recombinations between the different HERVs may cause genomic instability (Trombetta et al., 2016). For example, this mechanism may be responsible for at least 78 copy number variation cases encompassing known human genes (Campbell et al., 2014).

**Figure 1:**
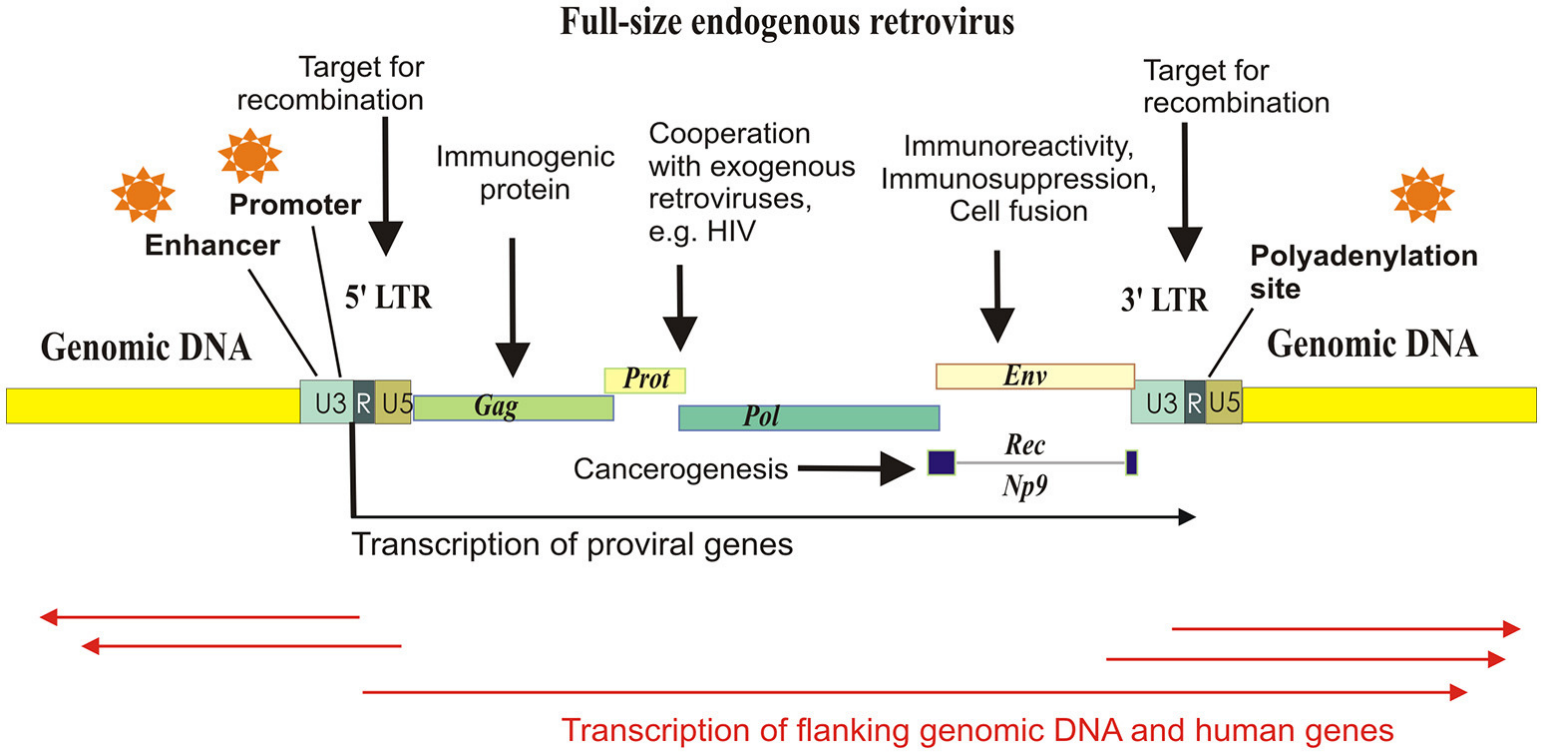
Schematized mechanisms of HERV influence on human gene expression and physiology. Major part of the HERV elements exist in the form of solitary LTRs, arisen due to homologous recombinations between the identical long terminal repeats flanking proviral genes Gag, Prot, Pol, and Env. HERV-K (HML-2) proviruses may have also additional gene termed “Rec” or “Np9,” depending on the retroviral subtype. Each LTR harbors polyadenylation signal, enhancer, and promoter elements, and can initiate transcription of the flanking genomic loci.

Most of the newly inserted HERVs harbor functional retroviral genes, such as those encoding for the reverse transcriptase/integrase, the structural polyprotein Gag and the envelope polyprotein Env, and the canonical function of an LTR is the regulation of retroviral expression. However, the LTRs may also drive the transcription of closely located genomic sequences and human genes (Buzdin et al., 2006a). In this minireview, we pay attention to the regulatory function of HERVs which donated multitude of functional sequences to the human genome.

## Structure of LTR and binding of nuclear proteins

Most of the HERVs exist in the form of solitary LTRs. The LTRs include promoter elements, enhancers, transcriptional factor binding sites, splice sites, and polyadenylation signals, and are thought to serve as the major transcriptional regulators of HERVs. LTRs specifically bind host cell nuclear proteins (Trubetskoy et al., 2002) and serve in the following five pathways of human transcriptional regulation: (i) LTRs may have enhancer/repressor activities (Domansky et al., 2000; Hughes and Coffin, 2004; Ruda et al., 2004; Suntsova et al., 2013); (ii) LTRs may be promoter active; (iii) LTR may provide polyadenylation sites to terminate read-through transcripts; (iv) LTRs may provide splice sites; (v) LTRs may regulate host genes by RNA interference (Gogvadze et al., 2009).

Mapping DNaseI hypersensitivity sites (DHS) is the method of choice for the high-throughput identification of the regulatory genomic regions. Similarly, transcription factor binding sites (TFBS) denote fragments of DNA with nuclear protein binding capacities (Ho et al., 2012). We combined investigation of both DHS and TFBS content of HERVs on a genomic scale (Garazha et al., 2015). To this end, we annotated all the genomic copies of HERVs and devised a bioinformatic algorithm mapping relevant TFBS and DHS features. For the entire set of HERVs, ~140,000 individual inserts (~19%) had at least one DHS and ~110,000 inserts (~15%)—at least one TFBS. Totally, there were identified ~155,000 and ~320,000 HERV-related DHS and TFBS, respectively (Garazha et al., 2015). This directly evidences potential implication of HERVs in the regulation of thousands of human genes. This is also in line with the previous finding that ~30% of all p53 binding sites localized by chromatin immunoprecipitation approach in the human genome fall within the HERV elements (Wang et al., 2007). Finally, as much as ~31.4% of all human transcription start sites were mapped within various transposable elements, including the HERVs (Faulkner et al., 2009).

All the 504 known HERV groups were characterized with regard to their TFBS content and showed very different results (available at http://herv.pparser.net/TotalStatistic.php). The families differed in their copy number, ranging from several copies as for the HERV-F, to more than 22,000 members as for the THE1B family. The total number of TFBS was also strikingly different—from zero (LTR5, LTR7A) to ~13,000 (MLT1K). The densities of TFBS also varied among the families. This is also important to quantitate absolute numbers of TFBS in each family. For example, the LTR12 family had the biggest proportion of TFBS-positive members and donated ~1,300 TFBS to the human DNA, whereas the family MLT1K contributed the greatest number of TFBS (~13,000), but had a small occurrence of TFBS-positive members. Interestingly, the TFBS and DHS tended to commonly appear in the same HERV elements. The probability that a particular element had DHS, was proportionate to the number of TFBS mapped herein (Garazha et al., 2015). Although, these findings provide clues for identification and functional annotation of multiple previously unknown human regulatory sequences, they are most likely still an underestimation of the HERV-generated TFBS pool. The repetitive nature of HERVs in many cases did not allow to directly attribute TFBS or DHS to any particular HERV element (Garazha et al., 2015).

Importantly, all the interrogated transcriptional factors had TFBS in the HERVs. This can explain extremely diverse and sometimes strongly tissue-specific influence of the different HERVs on the gene expression. For example, the LTR of the most recent HERV family HERV-K (HML-2) containing many human-specific and even polymorphic members, shows very high promoter and enhancer activities in the human germ cells and the corresponding tumors (seminoma), being transcriptionally silent in the other tissues (Domansky et al., 2000; Ruda et al., 2004). The promoter activity of the HERV-K (HML-2) inserts also provided the first evidence for the human specific antisense regulation of gene expression (Gogvadze et al., 2009). The human-specific LTRs located in the introns of genes *SLC4A8* and *IFT172* (for sodium bicarbonate cotransporter and intraflagellar transport protein 172, respectively) can *in vivo* generate transcripts that are reverse- complementary to the exons of those genes. Overexpression of the antisense transcripts resulted in approximately three- to four-fold decrease in mRNA levels for these genes (Gogvadze et al., 2009).

The HERVs can also provide polyadenylation signals for the regulation of gene expression. For example, mRNA for 8-kDa human protein similar to transcription factor GON4L is polyadenylated using the HERV-K (HML-2) LTR sequence (Baust et al., 2000). Another human transcription factor gene, *ZNF195*, utilizes the HERV-F LTR as the alternative polyadenylation site (Kjellman et al., 1999).

## Functional interplay of HERVs and human genome

Expression of HERVs is tightly controlled by the host cell because it may be deleterious. Even the physical presence of the repetitive sequences in the genome can generate genomic instability due to homologous recombination between the HERV elements. HERVs can bias normal gene regulatory networks (Suntsova et al., 2015; reviewed by Rebollo et al., 2012). Expression of HERV proteins may result in dangerous inflammatory or immunosuppressive effects (Cho et al., 2008). In mammals, endogenous retroviruses are transcriptionally repressed using the KRAB domain zinc finger proteins and their cofactor TRIM28, which recruit methylation machinery to HERV copies (Turelli et al., 2014). In embryonic cells, a zinc finger protein Yin Yang 1 may serve as another repressor of HERV transcription by suppressing promoter activities of the LTRs (Schlesinger et al., 2013). Besides DNA methylation, histone modification is considered an alternative mechanism of endogenous retroviral repression in embryonal stem cells with the proteins SETDB1 (methyltransferase responsible for H3K9 trimethylation) and H3K4 demethylase LSD1/KDM1A involved (reviewed by Rebollo et al., 2012).

APOBEC3 protein family has another function in suppression of HERVs and retroviruses. APOBEC3G (hA3G) inhibits the retroviruses by entering viral particles and inducing hypermutation of viral genome during reverse transcription, leading to G to A substitutions (Bae and Jung, 2014). In concert, the protein hA3F induces viral hypermutation by deaminating minus-strand of viral cDNA during reverse transcription (Bae and Jung, 2014). Taken together, these factors induce epigenetic silencing and hypermutation of HERVs. Indeed, the LTRs have a bigger mutation rate than the rest of non-coding fraction of the human genome (Romano et al., 2006).

Conversely, the content of TFBS among the HERVs decreases with their evolutionary age (Garazha et al., 2015). For the heavily mutated, highly diverged (>20%) HERV elements, this content is approximately six-fold lower compared to the top evolutionary young elements. This observation may suggest that genomic “domestication” of HERVs involved reformatting of the active TFBS profiles and their further “standardization” upon accumulation of mutations, until they get equilibrated with the rest of non-coding DNA (Garazha et al., 2015). However, this type of analysis can be biased by the higher fragmentation in the evolutionary older HERVs, because each fragment is considered as an independent element. Further studies are, therefore, needed to explore the TFBS accumulation trends in linkage to the evolutionary dynamics of the human genome.

Sometimes co-evolution with the human genome resulted in a recruitment of certain HERV regulatory modules by the host organism (Table 1). The best-known example is the acquisition of salivary expression of the carbohydrate digestive enzyme amylase from a HERV element inserted in the common ancestor of great apes (Ting et al., 1992).

**Table 1:** Implication of HERV transcriptional regulation in human physiology and pathology (selected examples).

**HERV element**	**Function**	**Mechanism**	**References**
**NORMAL PHYSIOLOGY**			
HERV-E	Expression of amylase genes *AMY1A, AMY1B, AMY1C* in salivary glands	Creates tissue-specific enhancer	Ting et al., 1992
HERV-K (HML-2)	Expression of proline dehydrogenase gene *PRODH* in hippocampus	Creates tissue-specific enhancer	Suntsova et al., 2013
HERV-H	Maintaining pluripotency in stem cells	Recruits transcriptional activators by initiating transcription of intergenic RNAs	Ohnuki et al., 2014
HUERS-P1	Maintaining pluripotency in stem cells	Promotes transcription of a non-coding RNA serving as a molecular sponge for miR let-7 microRNAs	Durruthy-Durruthy et al., 2015
MER39	Expression of Prolactin during pregnancy	Creates tissue-specific promoter	Emera et al., 2012
HERV9	Control of fetal and adult expression of globin locus	Recruits transcriptional factors to the downstream Beta-globin promoter	Tuan and Pi, 2014
HERV-K (HML-2)	Control of *SLC4A8* and *IFT172* gene expression	Promotes negative regulator antisense RNAs	Gogvadze et al., 2009
HERV-W	Fusion of trophoblast cells in placenta	Encodes protein Syncytin	Frendo et al., 2003
**CANCER**			
MaLR LTR	Survival of Hodgkin's lymphoma cells by upregulation of *CSF1R gene*	Creates alternative promoter	Lamprecht et al., 2010
HERV-K (HML-2)	Survival of Chronic Lymphocytic Leukemia cells	Encodes protein NP9 with possible oncogenic functions	Fischer et al., 2014
HERV-W	Tumor growth and metastasis via immunosuppression	Encodes protein Syncytin	Kassiotis, 2014
**INFECTIOUS DISEASES**			
HERV-L/HERV16	Suppression of Varicella zoster virus and HIV infection	Creates HLA Complex P5 gene	Crosslin et al., 2015
HERV-K	dUTPase activity for HIV life cycle	Encodes endogenous retroviral dUTPase	Mayer and Meese, 2003
**AUTOIMMUNE DISEASES**			
HERV-E	Possible role in promotion of systemic lupus erythematosus (SLE)	Encodes potentially immunogenic retroviral proteins	Wu et al., 2015
HERV-K10	Possible role in promotion of rheumatoid arthritis	Encodes potentially immunogenic retroviral protein HERV-K10 Gag	Nelson et al., 2014
HERV-W	Possible role in promotion of osteoarthritis	Encodes potentially immunogenic retroviral protein Syncytin	Bendiksen et al., 2014
HERV-K18	Possible role in promotion of osteoarthritis	Encodes potentially immunogenic retroviral proteins	Garcia-Montojo et al., 2013
**NEUROLOGICAL DISORDERS**			
Multiple HERVs	Possible role in promotion of multiple sclerosis	Encode potentially immunogenic proteins and induce autoimmunoreactiviry	Libbey et al., 2014; Manghera et al., 2015
HERV-W	Possible role in promotion of schizophrenia and bipolar disorder	Encodes potentially immunogenic retroviral protein Syncytin	Diem et al., 2012
HERV-K (HML-2)	Possible role in regulation of proline dehydrogenase in schizophrenia	Creates tissue-specific enhancer for gene *PRODH*	Suntsova et al., 2013
HERV-H	Induction of hypotonia and motor, language, and cognitive delays	Due to recombinations, mediate 3q13.2-q13.31 deletions	Shuvarikov et al., 2013

On the other hand, HERV-H is a family expressed preferentially in human embryonal stem cells. Surprisingly, these are the HERV-H LTRs that appeared to be the primary mediators of cell fate reprogramming using famous “Yamanaka cocktail” (by overexpressing OCT3/4, SOX2, and KLF4 proteins), due to regulatory HERV-H-driven intergenic non-coding RNAs that help to recruit the transcriptional activator genes by serving as the scaffold (Ohnuki et al., 2014). Another human long non-coding RNA (human pluripotency-associated transcript 5, *HPAT5*) derived from both a HERV element HUERS-P1 and an Alu retrotransposon, was shown to promote pluripotency by functioning as a molecular sponge for the let-7 family of microRNAs (Durruthy-Durruthy et al., 2015; Chuong et al., 2017).

The element MER39 forms an endometrium-specific promoter that regulates expression of Prolactin during pregnancy (Emera et al., 2012). The developmental switch from fetal to adult beta-globin gene expression in human is controlled by a copy of HERV9 element (Tuan and Pi, 2014). In hippocampus, transcription of gene *PRODH* is regulated human-specifically by a HERV-K (HML-2) LTR (Suntsova et al., 2013). *PRODH* metabolizes neuromediator molecules and has a strong implication in higher nervous activity and neurological disorders, and its deregulation might have an important impact on human evolution (Suntsova et al., 2013).

## HERV-mediated regulation of gene expression in pathology

### Proliferative disorders

Recent findings indicate that HERV-mediated control of gene expression may be involved in various human diseases including cancer (Kassiotis, 2014). The role of HERVs in cancer is most likely limited to regulation of gene expression (Hohn et al., 2013). The data from cancer genome sequencing identified over 180 somatic insertions caused by LINE-1 retrotransposon activity, vs. only a single integration of a short HERV fragment, most likely replicated due to microhomology-mediated DNA repair mechanism (Lee et al., 2012). Many HERVs are abnormally expressed in cancer. For instance, HERV-K (HML-2) elements are up to ~3,000 times overexpressed in germ cell tumors and in melanoma (Buzdin et al., 2006b; Schmitt et al., 2013). Upregulation of HERVs can be mediated by either biased content of the specific transcription factors or by disruption of the anti-retroviral suppression mechanisms, such as aberrant demethylation (Conti et al., 2016) and decreased expression of APOBEC3 proteins (Shepelin et al., 2016). HERVs, in turn, may promote cellular transformation by regulating downstream human genes. For example, a demethylated copy of MaLR LTR can act as an alternative promoter to transcriptionally derepress the gene *CSF1R*, encoding colony stimulating factor-1 receptor, which is linked with survival of the Hodgkin's lymphoma cells (Lamprecht et al., 2010). More examples can be found in the other specific reviews (Babaian and Mager, 2016; Gonzalez-Cao et al., 2016; Anwar et al., 2017).

### Infectious diseases

The evolution of human pathogens might generate mechanisms involving transcriptional interactions of endogenous and exogenous retroviruses. For example, in HIV-infected patients, the HERV-K (HML-2) proviruses are expressed in peripheral blood mononuclear cells at higher levels compared to the non-infected individuals (Bhardwaj et al., 2014). The antibodies against HERV-K (HML-2) Env protein in blood were proposed as the new biomarker of HIV-1 infection, because HIV-1 can upregulate expression of a fully N-glycosylated HERV-K (HML-2) envelope protein on the cell surface (Michaud et al., 2014). Moreover, the HERV-K (HML-2)-specific T-cells from the HIV-1 infected patients *in vitro* completely eliminated the human cells infected with a panel of globally diverse HIV isolates. The mechanism of HIV-1 induced activation of human transposable elements possibly involves the activity of an HIV-1 Tat protein (Jones et al., 2013). Recent studies showed that out of 91 annotated HERV-K (HML-2) proviruses, Tat could activate expression of 26 proviruses, silenced 12, and did not change the expression of the others (Gonzalez-Hernandez et al., 2014). In addition, HIV infection may cause transactivation of HERV-W elements with their Env genes and Syncytin (Uleri et al., 2014). However, a controversial data were reported on the presence of HERV-K (HML-2) viral particles in the plasma of HIV-infected patients—higher levels of HERV-K (HML-2) RNA were detected in the HIV patients from Uganda, but not from the USA (Li et al., 2013). Of note, the recent association study showed that susceptibility to infection with varicella zoster virus is linked with the non-coding gene HLA Complex P5 in the major histocompatibility complex. This gene is a copy of an endogenous retrovirus that may have a potential to suppress viral activity through indirect regulatory mechanisms. In previous studies, particular genetic variants of this region were associated with delay in development of AIDS in HIV-infected individuals (Crosslin et al., 2015).

### Autoimmunity

The biased expression of HERVs is considered as one of the triggers of autoimmune disorders (Suntsova et al., 2015), which is evidenced by increased proviral RNA levels (Ehlhardt et al., 2006) and anti-HERV protein antibodies in sera from several types of patients (Bannert and Kurth, 2004). Immune reactivity against ERV proteins can be experimentally induced in mice and non-human primates, evidencing that immunological tolerance to endogenous retroviral products is not complete (Kassiotis, 2014). The HERV overexpression may be linked with massive DNA hypomethylation as seen for T-cells in systemic lupus erythematosus (SLE) patients (Wu et al., 2015).

Compared to the normal controls, in the patients with rheumatoid arthritis, increased antibody response was detected against the HERV-K10 Gag protein (Nelson et al., 2014). HERV-W transcripts and protein isoforms of Syncytin were overexpressed in cartilage of osteoarthritis patients (Bendiksen et al., 2014). In osteoarthritis, the patient's individual disease severity index was correlated with the expression of HERV-K18 provirus (Garcia-Montojo et al., 2013). However, inflammatory diseases may be also associated with the decreased expression of HERVs (Table 1).

### Neurological diseases

Expression of HERVs may serve as the biomarker for various neurological diseases (Table 1). For example, the HERV expression may be inducible in human astrocytes and neurons under inflammatory conditions in an IFNγ-dependent manner (Manghera et al., 2015). For multiple sclerosis (MS), a hypothesis was proposed that HERV-encoded proteins can act as the powerful immune stimulators inducing disease progression following neurodegeneration (Libbey et al., 2014). Indeed, genetic variants in some genes restricting retroviral infections were statistically linked with the risk of getting MS, as shown for the TRIM5, TRIM22, and BST2 genes (Nexo et al., 2013).

The abnormally high levels of the HERV-W Env gene product were detected in the plasma of the patients with schizophrenia and bipolar disorder (Diem et al., 2012), and in the active lesions in multiple sclerosis (van Horssen et al., 2016) and in the biopsies from the chronic inflammatory demyelinating polyradiculoneuropathies (Faucard et al., 2016). The increased expression of endogenous HERV-K (HML-2) proviral Env gene, in turn, may contribute to the development of amyotrophic lateral sclerosis by inducing neurodegeneration (Li et al., 2015). Finally, HERVs may also cause neurological disorders due to HERV-linked genomic rearrangements (Table 1). The human-specific enhancer activity of a HERV-K (HML-2) provirus on schizophrenia-associated gene *PRODH* may be another active mechanism of HERV involvement in schizophrenia (Suntsova et al., 2013). Recently, a link was discovered between schizophrenia risk and the complement C4 system (Sekar et al., 2016). The individuals having a polymorphic HERV intronic insertion have elevated C4 expression, which in turn may cause neuronal synapse over-pruning, a phenotype that is associated with schizophrenia. Although this evidence is still indirect, this case is intriguing in light of previous observations of an association between schizophrenia and elevated ERV transcriptional activity (Chuong et al., 2017).

## Conclusions

Taken together, these findings suggest that at the early stages after insertion, the HERV is treated by the host cells as a foreign genetic element, and is likely to be suppressed by the targeted methylation and mutations. However, at the later stages, when significant number of mutations has been already accumulated and when the retroviral genes are broken, the regulatory potential of a HERV may be released and recruited to modify the genomic balance of transcription factor binding sites. This process goes together with further accumulation and selection of mutations, which reshape the regulatory landscape of the human DNA. However, developmental reprogramming, stress or pathological conditions like cancer, inflammation and infectious diseases, can remove the blocks limiting expression and HERV-mediated host gene regulation. This, in turn, can dramatically alter the gene expression equilibrium and shift it to a newer state, thus further exacerbating the stressful or unstable situation.

## Author contributions

All authors listed have made substantial, direct and intellectual contributions to the work, and approved it for publication.

### Conflict of interest statement

The authors declare that the research was conducted in the absence of any commercial or financial relationships that could be construed as a potential conflict of interest.
